# Using Statistical Parametric Mapping as a statistical method for more detailed insights in swimming: a systematic review

**DOI:** 10.3389/fphys.2023.1213151

**Published:** 2023-06-29

**Authors:** Jorge E. Morais, Tiago M. Barbosa, Tomohiro Gonjo, Daniel A. Marinho

**Affiliations:** ^1^ Instituto Politécnico de Bragança, Department of Sports Sciences, Bragança, Portugal; ^2^ Research Center in Sports Health and Human Development (CIDESD), Covilhã, Portugal; ^3^ School of Energy, Geoscience, Infrastructure, and Society, Institute for Life and Earth Sciences, Heriot-Watt University, Edinburgh, United Kingdom; ^4^ University of Beira Interior, Department of Sports Sciences, Covilhã, Portugal

**Keywords:** swimming, training, exercise, continuous analysis, sensitive outputs

## Abstract

Swimming is a time-based sport and hence strongly dependent from velocity. Most studies about swimming refer to velocity as discrete variable, i.e., 0-D (no time dimension). However, it was argued that using swimming velocity as a continuous variable (1-D, with time dimension) with Statistical Parametric Mapping (SPM) can bring deeper and detailed insights about swimming performance. Therefore, the aim of this study was to perform a systematic review about the current body of knowledge of using Statistical Parametric Mapping in a swimming context. The Preferred Reporting Items for Systematic Reviews and Meta-Analyses (PRISMA) guidelines were used to identify relevant articles. After screening, nine articles related to Statistical Parametric Mapping (SPM) analysis in swimming were retained for synthesis. Results showed that four articles (44.4%) aimed to understand the kinematics, isokinetic joint torque or electromyographic (EMG) pattern of the swimmer’s shoulder either on land or during front crawl trials. Two articles (22.2%) focused on understanding the swimming velocity while performing the breaststroke stroke. One article (11.1%) analyzed the swimmers’ propulsion at front-crawl stroke, another one (11.1%) compared swimming velocity during a complete stroke cycle in young swimmers of both sexes as a discrete variable and as a continuous variable. Also, one article (11.1%) analyzed the underwater undulatory velocity. In an EMG context, some findings verified in SPM are not possible to be discovered with traditional 0-D statistical methods. Studies about swimming velocity (breaststroke, freestyle, and underwater undulatory velocity) and propulsion (front-crawl) also highlighted the SPM advantages in comparison to traditional statistical methods. By using SPM, researchers were able to verify specifically where within the stroke cycle significant differences were found. Therefore, coaches can get more detailed information to design specific training drills to overcome hypothetical handicaps.

## 1 Introduction

The main aim of sports science is to identify, develop and refine strategies to improve performance (Fullagar et al., 2019). Sport science includes a wide variety of scientific fields (e.g., physiology, biomechanics, strength and conditioning, motor control, nutrition, psychology, and performance analysis) from which researchers, coaches and practitioners aim to excel the performance development (Morais et al., 2018; Marinho et al., 2020; Sokołowski et al., 2022). Based on the multifactorial and holistic phenomenon that performance is, sports science community seeks to maximize the athletes’ strengths and overcome their weaknesses. This will have a meaningful effect on their performance enhancement (Logue et al., 2018) and injury prevention (Faude et al., 2017).

Competitive swimming is a time-based sport; therefore, researchers and coaches are keen on understanding how to improve swimming velocity (Morais et al., 2018; Olstad et al., 2022). Most research about swimming velocity measures this variable as an average value (i.e., discrete variable—0-D, no time dimension). For instance, in race analysis, swimming velocity is usually measured lap-by-lap (Veiga and Roig, 2016; Morais et al., 2020) or based on different sections of each lap (i.e., velocity achieved at different distances) (Simbana-Escobar et al., 2018; Morais et al., 2022b). In experimental studies, it is usually measured based on maximal (Morais et al., 2016), sub-maximal (de Matos et al., 2022), or controlled trials (Gonjo et al., 2019). Notwithstanding, even when instantaneous velocity is measured, authors report the average value of the entire stroke cycle(s) measured (Silva et al., 2019; Takagi et al., 2021). However, this method provides only insights into the swimming velocity achieved during the entire stroke cycle or the average of a combination of stroke cycles.

Swimming velocity is described as a periodic signal that depends on the net balance between propulsion and drag forces acting on the swimmers (Barbosa et al., 2010). This determines the body´s acceleration and hence the swimming velocity changes:

$$a=\left(P-D\right)/m$$ (1)

Where *a* refers to acceleration (in m/s^2^), *P* is the propulsion (in N), *D* is the hydrodynamic resistive force (in N), and *m* (kg) refers to the swimmer’s body mass. This equation shows that the swimmer accelerates when the magnitude of P exceeds the magnitude of D, and when D is greater, the swimmer decelerates. Using swimming velocity as a discrete variable (0-D, with no time dimension) will give only an overall perspective of the entire stroke cycle or a combination of several stroke cycles, which disregards the complexity of the swimming velocity pattern that repeats acceleration and deceleration (Morais et al., 2016; Silva et al., 2019). On the other hand, continuous analysis (1-D, with time dimension) has been shown to present significant advantages by providing more accurate and sensitive details whenever continuous time-series are used (Pataky, 2010). In swimming, and as far as our understanding goes Principal Component Analysis (PCA) or Statistical Parametric Mapping (SPM) have increased in popularity over the years in continuous time-series analysis (Burkhardt et al., 2020; Gourgoulis and Nikodelis, 2022). In the case of SPM, this statistical method exploits the use of random field theory to perform topological inference by directly mapping the conventional Gaussian distribution to smooth n-dimensional data (Pataky, 2010). Hence, SPM can be employed to keep the integrity and sensitivity of time-series data. This statistical method is increasingly used in sports sciences, contributing to more detailed movement analyses in biomechanical and performance analyses (Warmenhoven et al., 2018; Bertozzi et al., 2022). One can argue that this occurs mainly due to the open source spm1d code (www.spm1d.org). Todd Pataky and co-workers have introduced SPM in the biomechanics and human movement science community, for instance in univariate or multivariate time series data regarding kinematics, kinetics, electromyography, etc.) (Pataky, 2012). SPM presents strong advantages for biomechanists by maintaining the originally sampled time series. Since kinematic, kinetic or electromyographic (EMG) time series can be complex, it can be difficult to objectively specify an a-priori method for analysis. Therefore, by using SPM for time series data, the statistical result is still a time series (e.g., a time series of t-values) and allows for better interpretation of data (Serrien et al., 2019). Movements need to be normalized by defining the start and end points. Afterwards, SPM will give the outputs required based on every percentual point between 0% and 100% (Pataky et al., 2017).

However, the literature lacks a summary about the kind of outputs that SPM can provide in a swimming context where swimming velocity is highly studied. As all four swim strokes present key-moments within the stroke cycle (both in the upper- and lower-limbs) (Barbosa et al., 2011), one can argue that more specific and accurate outputs can be retrieved and provided to both swimmers and coaches. That is, instead of showcasing the overall trend, differences in specific phases of a given movement can be addressed. For instance, in kayaking, it was shown that during the sprint, joint kinematics significantly changed over time at the shoulder, trunk, and hip levels (Bertozzi et al., 2022). The authors noted that, for the case of the shoulder, SPM showed a decrement of the shoulder elevation at the end of the sprint between around the 50% of the cycle and about 80%, showing how fatigue affects kayaking kinematics (Bertozzi et al., 2022). In the case of swimming, specifically in breaststroke, it was noted that elite swimmers showed a faster velocity between the 20% and the 40% of the glide segment after the start (Gonjo and Olstad, 2021). These examples demonstrate that SPM can provide information about where the strength and weakness of different groups, or different conditions, exist within a given time-frame. Therefore, performing a systematic review about the current body of knowledge of using SPM in a swimming context, may provide researchers and coaches with more in-detail information about swimming performance.

## 2 Methods

### 2.1 Literature search and article selection

**FIGURE 1:**
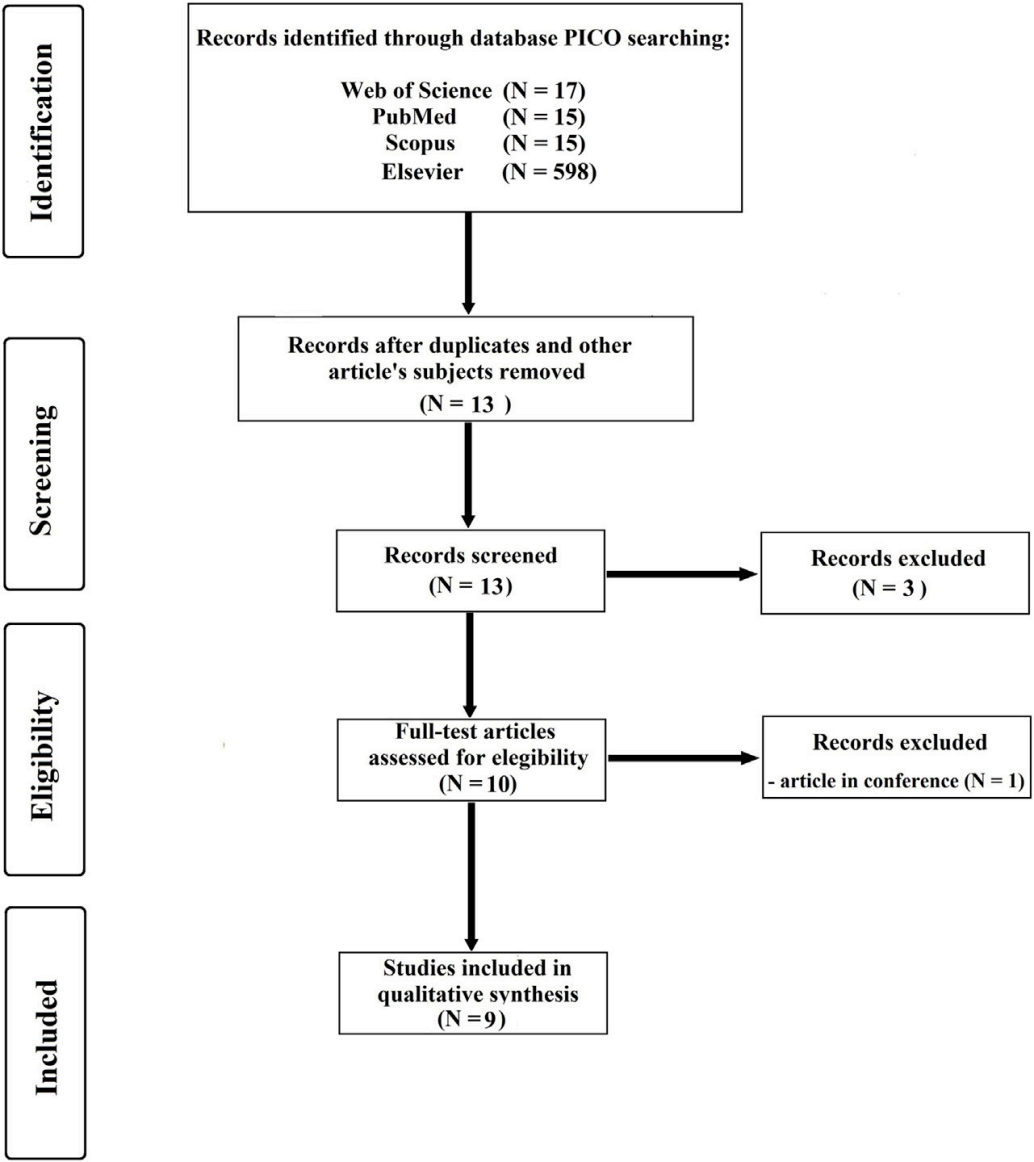
Summary of PRISMA flow for search strategy.

The Web of Science, PubMed, Scopus, and Google Scholar databases were searched to identify studies that aimed to use Statistical Parametric Mapping in swimming. As an initial search strategy, the title, abstract and keyword of the text were first identified and read carefully for a first scan and selection of the journal’s articles. The Boolean search method (including AND/OR) was used to identify the literature containing keywords and terms related to the topic (Waffenschmidt et al., 2013). The following were chosen as inclusion criteria: i) written in English; ii) published in a peer-reviewed journal; iii) related to competitive swimming, and; iv) healthy and able-bodied swimmers. After deleting all duplicated and unrelated articles, nine articles were included. The final search was carried out on 12 June 2023. Table 1 presents the PI(E)CO search strategy used (P—patient, problem, or population; I—intervention; E—exposure; C—comparison, control, or comparator; O—outcomes). Figure 1 depicts the PRISMA flow diagram for identifying, screening, checking eligibility, and inclusion of the articles.

**TABLE 1:** PI(E)CO (P—patient, problem or population; I—intervention; E—exposure; C—comparison, control, or comparator; O—outcomes) search strategy.

Population	Intervention or exposure	Comparison (design)	Outcome
Swimmer^a^	Biomechanics	Cross-sectional	Performance
Athlete^a^	Kinematics	Longitudinal	Velocity/speed
Child^a^	Kinetics	Experimental	Time-series
Boy^a^	Swimming	Randomized control trial	Continuous analysis
Girl^a^			Statistical Parametric Mapping
Male			Variation
Female			Fluctuation

^a^ truncation to retrieve words with different endings.

### 2.2 Quality assessment

The Downs and Black Quality Assessment Checklist was used to assess the quality of each article (Downs and Black, 1998). This was based on the following criteria: i) reporting; ii) external validity; iii) internal validity (bias and confounding); and iv) power. The original version has 27 items with a maximum score of 32 points. Adaptations were made to the original version, according to the focus of included studies and the previously modified versions. For instance, items 4, 8, 9, 14, 15, 17, 19 and 22 to 26 were excluded when not applicable to the study design as described by others (Hébert-Losier et al., 2014; Santos et al., 2021), which resulted in the maximum score of 17 points. Quality was classified as: i) low if scored ≤50%; ii) good if scored between 51% and 75%, and; iii) excellent if scored >75% (Sarmento et al., 2018). The agreement between two independent reviewers was calculated with the Cohen’s Kappa coefficient, and interpreted as: i) no agreement if K < 0; ii) poor agreement if 0 < K < 0.19; iii) fair agreement if 0.20 < K < 0.39; iv) moderate agreement if 0.40 < K < 0.59; v) substantial agreement if 0.60 < K < 0.79; and vi) almost perfect agreement if 0.80 < K < 1.00 (Landis and Koch, 1977).

## 3 Results

### 3.1 Quality assessment

The articles included in the final review stage had a score of 11.75 ± 1.54 points (69.12%—scored as good quality). The primary reason for some articles not achieving a higher score was mainly due to the non-inclusion of information related to the statistical power calculation. Agreement between evaluators revealed an almost perfect agreement (K = 0.96, *p* < 0.001, 95% confidence intervals: 0.91–1.01).

### 3.2 Participants’ demographics

Table 2 presents the sample demographics for each study. This includes the sample size, participants’ age, body mass, height, years of experience, pool length where data was collected, the type of trial or event, and the participants’ FINA points that characterize their competitive level. Most studies (N = 6, 66.7%) (Martens et al., 2016; Gaudet et al., 2018a; 2018b; Blache et al., 2018; Gonjo and Olstad, 2021; Gourgoulis and Nikodelis, 2022) evaluated adult swimmers, and three studies (33.3%) investigated young swimmers (Ruiz-Navarro et al., 2021; Morais et al., 2022c; 2023). Five studies (55.6%) (Martens et al., 2016; Blache et al., 2018; Gonjo and Olstad, 2021; Morais et al., 2022c; Gourgoulis and Nikodelis, 2022) evaluated only male swimmers, and four studies (44.4%) included males and females concurrently (Gaudet et al., 2018a; 2018b; Ruiz-Navarro et al., 2021; Morais et al., 2023). Also, six studies (66.7%) used a swimming trial to collect and analyze data (Martens et al., 2016; Gonjo and Olstad, 2021; Ruiz-Navarro et al., 2021; Morais et al., 2022c; 2023; Gourgoulis and Nikodelis, 2022), while three (33.3%) did not request the swimmers to perform any swimming trials (Gaudet et al., 2018a; 2018b; Blache et al., 2018).

**TABLE 2:** Summary of the sample demographics of each study included for analysis. n.a.—not applicable (i.e., not reported).

Source	Sample	Years of experience	Data collection environment	Race/trial event	FINA points
Blache et al. (2018)^a^	Four groups	G0: None	Land	n.a	French federation quotation
	G0: N = 11 males; 23.2 ± 1.7 years; 1.73 ± 0.10 m of height; 68.2 ± 13.5 kg of body mass	G1: 10.5 ± 2.3 years			G0: 0.0 ± 0.0
	G1: N = 11 males; 17.0 ± 1.0 years; 1.86 ± 0.07 m of height; 71.1 ± 7.1 kg of body mass	G1: 13.9 ± 5.1 years			G1: 1192 ± 59
	G2: N = 10 males; 21.9 ± 2.2 years; 1.84 ± 0.06 m of height; 76.9 ± 8.7 kg of body mass	G1: 13.7 ± 4.7 years			G1: 1248 ± 83
	G3: N = 10 males; 20.8 ± 4.4 years; 1.76 ± 0.07 m of height; 69.2 ± 11.1 kg of body mass				G1: 1009 ± 138
Gaudet et al. (2018a)	11 males and 14 females: 22.8 ± 4.4 years; 174 ± 9.0 cm of height; 68.7 ± 10.6 kg of body mass. Active adults: N = 17; competitive swimmers: N = 8	n.a	Land	n.a	n.a
Gaudet et al. (2018b)	N = 11 males and 13 females: 22.6 ± 4.3 years; 174 ± 9.1 cm of height; 69.4 ± 11.0 kg of body mass. Active adults: N = 17; competitive swimmers: N = 7	Active adults: recreational physical activities at least two times per week for the past 3 years (until data collection). Competitive swimmers: 5 years and swam at least 7 times per week in addition to resistance training two times per week	Land	n.a	n.a
Gonjo and Olstad (2021)	N = 14 males Elite: N = 7; 20.0 ± 2.4 years; 1.85 ± 0.5 m of height; 82.3 ± 5.2 kg of body mass	n.a	Water: 25 m swimming pool	100 m breaststroke	Elite: 772.1 ± 35.2 at the 100 m breaststroke event
	Sub-elite: N = 7; 17.7 ± 0.9 years; 1.81 ± 0.4 m of height; 75.1 ± 4.6 kg of body mass				Sub-elite: 610.6 ± 24.7 at the 100 m breaststroke
Gourgoulis and Nikodelis (2022)	N = 9 males; 21.57 ± 4.20 years; 1.79 ± 0.07 m of height; 75.60 ± 6.11 kg of body mass		Water: 25 m swimming pool	One maximal and one sub-maximal trial of 25 m	688.80 ± 131.22 at the 100 m breaststroke event in long course meter pool
Martens et al. (2016)	N = 15 males; 21.26 ± 2.24 years; 186.55 ± 5.50 cm of height; 79.10 ± 7.98 kg of body mass	11.93 ± 3.24 years	Water: 25 m swimming pool	25 m trial at front-crawl	634.13 ± 68.98 at the 100 m freestyle event
Morais et al. (2022c)	N = 22 males Juniors: N = 12; 16.35 ± 0.74 years; 177.42 ± 5.14 cm of height; 70.64 ± 5.65 kg of body mass; 182.96 ± 9.15 cm of arm span	More than 5 years of experience	Water: 25 m swimming pool	25 m trial at front-crawl	Juniors: 572.17 ± 67.32 at the 100 m freestyle event in short-course meter
	Juveniles: N = 10; 15.40 ± 0.32 years; 176.30 ± 6.46 cm of height; 66.88 ± 8.14 kg of body mass; 182.50 ± 7.74 cm of arm span				Juveniles: 560.30 ± 43.72 at the 100 m freestyle event in short-course meter
Morais et al. (2023)	Boys: N = 60; 12.91 ± 0.86 years; 162.00 ± 11.07 cm of height; 51.23 ± 9.01 kg of body mass; 167.45 ± 11.07 cm of arm span	More than 3 years of experience	Water: 25 m	25 m trial at front-crawl	Boys: 283.49 ± 85.18 at the 100 m freestyle in short-course meter
	Girls: N = 60; 12.46 ± 0.94 years; 158.42 ± 5.87 cm of height; 49.51 ± 7.22 kg of body mass; 161.09 ± 6.10 cm of arm span				Girls: 315.05 ± 85.72 at the 100 m freestyle in short-course meter
Ruiz-Navarro et al. (2021)	N = 17	At least 2 years with five training sessions per week	Water: 12.50 m	10 m trials of underwater undulatory swimming	n.a
	Boys: N = 10; 11.6 ± 0.2 years; 1.47 ± 0.01 m of height; 39.2 ± 1.4 kg of body mass				
	Girls: N = 7; 10.6 ± 0.4 years; 1.45 ± 0.04 m of height; 38.2 ± 3.6 kg of body mass				

^a^ – G0: practicing no sports; G1: adolescent elite swimmers; G2: adult elite swimmers; G3: club-level adult swimmers.

### 3.3 Purpose, variables, and main outcomes

Table 3 presents information about the purpose, variables measured, SPM test used, and main outcomes. Four studies (44.4%) aimed to understand the kinematics, isokinetic joint torque or EMG pattern of the swimmer’s shoulder either on land (Gaudet et al., 2018a; 2018b; Blache et al., 2018) or during front crawl trials (Martens et al., 2016). Among the four, one study also focused on the abdominal muscle EMG pattern in front crawl (Martens et al., 2016). Overall, it was shown that: i) swimmers tend to adapt their scapular internal rotation on land mainly due to the accumulation of years of swimming practice at high level (Blache et al., 2018). The subscapularis and serratus anterior muscles play a key role in the stability of the shoulder joint (Gaudet et al., 2018a). A decrease in peak torque throughout the repetitions observed in a set of muscles (namely, pectoralis major, middle deltoid, and periscapular and rotator cuff) suggested signs of fatigue (Gaudet et al., 2018b). At the front crawl stroke, several activation patterns were noted for deltoideus medialis and rectus abdominis muscle recruitment (Martens et al., 2016).

**TABLE 3:** Summary of the purpose, variables measured, and main outcomes. n.a.—not applicable (i.e., not reported).

Source	Purpose	Variables	SPM test	Outcomes
Blache et al. (2018)	To: i) describe and compare scapular kinematics between three groups of swimmers of different levels and a group of non-swimmers; ii) assess whether swimming practice alters the asymmetries in scapular kinematics between the dominant and nondominant sides, both during unilateral arm raising and lowering in the scapular plane	Angles related to	• One-Way ANOVA SPM on two repeated measures	• Swimmers presented forward posture evidenced by clavicular protraction
		• Shoulder proctration/rectration with respect to the humerothoracic elevation		• Bilateral differences in scapular upward rotation were eliminated suggesting safe adaptations of their non-dominant shoulder
		• Internal/external rotation of the scapula with respect to the humerothoracic elevation		• Adaptations in scapular internal rotation may be due to the accumulation of years of practice at high level
		• Downward/upward rotation of the scapula with respect to the humerothoracic elevation		
Gaudet et al. (2018a)	To determine if there were differences in the peak EMG activity and in the recruitment pattern between the two types of movement (internal versus external rotation), two velocities (60° vs. 240°/s), and the type of contraction (concentric vs. eccentric) for the shoulder girdle muscles	EMG activation of a set of muscles while performing a maximum concentric and eccentric shoulder internal and external rotations at velocities of 60° and 240°/s	• Three-way ANOVA SPM.	• Subscapularis and serratus anterior play an important role in the stability of the shoulder joint in both internal and external rotations
		• Pectoralis		• Rapid eccentric contractions may teach the athletes faster recruitment of the shoulder girdle muscles while developing higher moment force
		• Latissimus dorsi		
		• Middle deltoid		
		• Posterior deltoid		
		• Upper trapezius		
		• Middle trapezius		
		• Lower trapezius		
		• Serratus anterior		
		• Supraspinatus		
		• Infraspinatus		
		• Subscapularis		
Gaudet et al. (2018b)	To assess the effect of a fatiguing protocol on shoulder strength and muscle activity during repeated maximal internal-external isokinetic shoulder rotations	EMG activation of a set of muscles while performing a fatigue protocol of a set of 50 repetitions, each consisting of maximum concentric shoulder internal rotation and external rotation efforts at a velocity of 240°/s	• One-Way ANOVA SPM.	• A significant decrease in MDF was noted in the pectoralis, middle deltoid, upper, middle and lower trapezius, infraspinatus and subscapularis muscles
		• Pectoralis		• This indicates a sign of fatigue as confirmed by a decrease in peak torque throughout the repetitions
		• Latissimus dorsi		• Present results can be used to improve shoulder injury prevention and rehabilitation programs of swimming and overhead athletes
		• Middle deltoid		
		• Posterior deltoid		
		• Upper trapezius		
		• Middle trapezius		
		• Lower trapezius		
		• Serratus anterior		
		• Supraspinatus		
		• Infraspinatus		
		• Subscapularis		
Gonjo and Olstad (2021)	To investigate differences throughout 100 m breaststroke between elite and sub-elite swimmers using time-series velocity data	In each one of four laps	• T-test SPM (between groups comparison)	• Elite swimmers are characterized by larger clean-swimming and gliding velocity
		• Gliding velocity		• Specifically, at the beginning of the glide segment and most of the clean swimming (except for the beginning of this segment)
		• Pull-out velocity		• No differences in the pull-out and at the beginning of the clean-swimming phases indicated that techniques to produce fast clean swimming velocity does not necessarily guarantee fast underwater pullout and transition stroke velocity
		• Clean swimming velocity		
Gourgoulis and Nikodelis (2022)	To investigate any possible modifications in the stroke kinematics during breaststroke swimming with maximal and sub-maximal intensity	For X, Y, and Z-axis the	• T-test paired samples SPM.	• The main discriminating factors between maximal and sub-maximal breaststroke swimming seems were: i) the adjustment of the duration of the glide phase, and; ii) the velocity of the hands during the recovery phase
		• Center of mass velocity		
		• Hand velocity		
Martens et al. (2016)	To: i) investigate inter-individual variability in front crawl swimming using variability measures, and; ii) determine if EMG sub patterns could be found using key features selected with both qualitative and quantitative classification strategies in a cluster analysis	Maximal voluntary isometric contraction	• Two-tailed two sample T-test SPM, and a ANOVA SPM.	• In front crawl swimming there is not one general activation pattern for deltoideus medialis and rectus abdominis
		• Left deltoideus medialis		• Nonetheless, several sub-patterns were verified. These were statistically different from each other during specific parts of the stroke cycle
		• Right deltoideus medialis		• This was mainly due to differences in amplitude
		• Left rectus abdominis		
		• Right rectus abdominis		
Morais et al. (2022c)	To: i) compare swimming velocity and a set of kinematical variables between junior and juvenile swimmers, and; ii) compare the propulsion outputs through discrete and continuous analyses (SPM) between junior and juvenile swimmers for each upper limb (i.e., dominant and non-dominant)	• Swimming velocity	• Two-tailed independent sample T-tests SPM.	• Juniors were significantly faster than juvenile swimmers
		• Intra-cyclic fluctuation of swimming velocity		• Juniors also presented greater values of propulsion than their juvenile counterparts (but not significantly)
		• Stroke frequency		• This indicates that immediately after the propulsion phase, swimmers must adopt a position that allows them to significantly reduce drag
		• Stroke length		• Statistical parametric mapping provides coaches and swimmers with deeper insights about swimmers’ propulsion during the entire arm-pull
		• Mean propulsion (both upper-limbs)		
		• Intra-cyclic fluctuation of propulsion (both upper-limbs)		
Morais et al. (2023)	To compare swimming speed during a complete stroke cycle in young swimmers of both sexes as a discrete variable and as a continuous variable (SPM)	• Body mass	• Two-way SPM ANOVA.	• dv analysis gives an overall overview about the swimmers’ displacement, where less dv is related to better performances
		• Height		• SPM analysis allowed a more sensitive analysis of the swimming speed-time series by pinpointing in which key-moment of the cycle differences were observed
		• Arm span		• In boys and girls, all three tiers differed from each other
		• Speed		• In tier comparison between sexes, significant differences were only found in tier #3 (poorer performers)
		• dv		
		• Stroke frequency		
		• Stroke length		
Ruiz-Navarro et al. (2021)	To evaluate the performance and kinematics of underwater undulatory velocity changes after a period of training in young swimmers	Pre vs. post-test	• Paired samples T-test SPM.	• After a period of 7 weeks, swimmers improved their underwater undulatory velocity and gliding performance
		• Average kick velocity-time curve		• The upbeat execution was the key-factor for the underwater undulatory speed enhancement
		• Underwater gliding velocity-time curve		• Gliding performance may have improved based on the ability to maintain a better hydrodynamic position (which may have reduced drag)

SPM, statistical parametric mapping; EMG, electromyography; MDF, median frequency decline in EMG, signals; dv—intra-cyclic variation of swimming speed.

Two studies (22.2%) focused on understanding the swimming velocity while performing the breaststroke stroke (Gonjo and Olstad, 2021; Gourgoulis and Nikodelis, 2022). It was discovered that: i) elite swimmers presented a faster velocity, mainly at the beginning of the glide segment and most of the clean swimming, than their sub-elite counterparts (Gonjo and Olstad, 2021), and; ii) the adjustment of the duration of the glide phase, and the velocity of the hands during the recovery phase were the main discriminant factors that distinguished maximal from sub-maximal trials (Gourgoulis and Nikodelis, 2022).

One study (11.1%) analyzed the swimmers’ propulsion in front-crawl stroke (Morais et al., 2022c). It was observed that despite junior swimmers presented greater values of propulsion than their juvenile counterparts in both upper limbs, this was not significant in any moment of the stroke cycle (Morais et al., 2022c). Another one (11.1%) compared swimming velocity during a complete stroke cycle in young swimmers of both sexes as a discrete variable and as a continuous variable (SPM) (Morais et al., 2023). Based on the SPM outputs, significant sex and tier effects and a significant sex*tier interaction in some moments of the stroke cycle were noted in young swimmers. Thus, it made it possible to understand where within the stroke cycle boys and girls of different tiers differed. Finally, one study (11.1%) analyzed the underwater undulatory velocity (Ruiz-Navarro et al., 2021). It was exhibited that the upbeat execution was the key-factor for the underwater undulatory velocity enhancement (Ruiz-Navarro et al., 2021). As the underwater undulatory velocity was significantly faster during the complete execution of the upbeat, the authors argued that the improvements verified in the underwater undulatory velocity were mostly produced by a better execution of the upbeat. That is, between the pre- and post-test swimmers were able to minimize the velocity decrease in the upbeat execution (Ruiz-Navarro et al., 2021).

## 4 Discussion

The aim of this study was to review the current body of work on the application of SPM in swimming. Nine articles were related to swimming and therefore included for analysis. Overall, four studies were related to the EMG pattern of shoulder muscles, two studies with the breaststroke stroke (swimming velocity), one study with the front-crawl stroke (swimming velocity), one study with the front-crawl stroke (propulsion), and one study with underwater undulatory velocity.

Our analysis revealed that most studies using SPM in swimming were related to the EMG pattern of the shoulder muscles (Martens et al., 2016; Gaudet et al., 2018a; 2018b; Blache et al., 2018). Research with EMG signals aims to monitor muscle functions and coordination in different movements or postures (Clarys and Cabri, 1993). As all four swim strokes require different muscles, researchers and coaches can design dry-land-specific training sets for enhancing a given muscle strength aiming to improve performance and diminish the risk of injury (Crowley et al., 2018; Batalha et al., 2020). Despite the importance of monitoring the muscular activities in each swimming stroke, among the four EMG studies reviewed in the present study, only one study conducted SPM analysis during swimming performance (Martens et al., 2016). Remaining ones referred to: i) the unilateral arm raising/lowering in the scapular plane (with the thumb pointed in the upward direction) (Blache et al., 2018), and; ii) concentric and eccentric internal—external shoulder rotations (Gaudet et al., 2018a; 2018b), all of which were non-swimming activity on land. Thus, at least until now, studies using SPM in an EMG context are more focused on general movements rather than specifically focusing on swim stroke movements.

In the study by Martens et al. (2016), the authors aimed to first verify the hypothetical formation of several sub-patterns of muscle activity through cluster analysis. Afterwards, they used SPM to compare the muscle activity between the clusters formed. The authors noted that, with two, three, or four clusters, a significant difference was noted between clusters. For the deltoid medialis, SPM revealed that the exit and early recovery phases were the most discriminating phases. As for the rectus abdominis, the transition from pull to push phase, and the recovery phase were the ones that better discriminated the clusters (Martens et al., 2016). Thus, the authors claimed that future studies about EMG (at least in front-crawl) could specifically define the key features to be analyzed (Martens et al., 2016). Others requested the swimmers to perform a dry-land movement that consisted in raising and lowering the arm in the scapular plane (Blache et al., 2018). The authors indicated that a significant group effect was only noted for the shoulder protraction/retraction between 30° and 120° of humeral elevation during the arm raising and lowering. Shoulders were more retracted in the control group than in remaining ones. As for the scapula internal/external rotation a significant group effect was noted between the 67° and 116° of humeral elevation during arm raising, and between the 81° and 54° during arm lowering. The scapular positioning in internal rotation was similar in all groups, except in the adult elite. This presented an increased scapular internal rotation in comparison to the remaining groups. As for the scapula upward/downward rotation a significant interaction group*laterality effect was noted between the 74° and 104° of humeral elevation during arm raising. Overall, a significant imbalance was only noted in the control group. Therefore, the authors concluded that: i) swimming practice caused an increased clavicular protraction and removed bilateral differences in scapular upward rotation during scaption, and; ii) alterations in scapular internal rotation positioning were related to the swimming practice level, i.e., more years of practice were related to greater alterations (Blache et al., 2018).

Another research group aimed to analyze the muscle recruitment pattern (11 muscles) and the specific evolution of fatigue of the shoulder (Gaudet et al., 2018a; 2018b). Regarding muscle recruitment, SPM revealed significant effects of the velocity and type of contraction in all muscles analyzed (see table 3 for the muscles analyzed) (Gaudet et al., 2018a). Based on the concentric/eccentric contraction significant differences in the EMG activity were noted for the pectoralis major, latissimus dorsi and subscapularis muscles during internal rotation. As for the external rotation all showed significant differences but the pectoralis major. Specifically, significant differences were noted between the −25% to 15% of normalized cycle (i.e., pre-activation region) for the eccentric contraction. As for the concentric contraction, EMG activity was significantly higher for most muscles during 15%–30% of the normalized cycle (Gaudet et al., 2018a). By using SPM, the authors concluded that: i) supraspinatus and infraspinatus muscles behave more as prime movers than stabilizers, and SPM analysis provided deeper insights into the recruitment pattern differences between concentric and eccentric conditions (Gaudet et al., 2018a).

When aiming to understand the fatigue accumulation in the shoulder muscles by SPM, Gaudet et al. (2018b) investigated the effect of fatigue on the instantaneous median frequency for the pectoralis, middle deltoid, upper, middle and lower trapezius, infraspinatus and subscapularis muscles using a 50-repetitions fatigue test in the maximum shoulder internal and external concentric rotation. The authors divided the 50 repetitions in five blocks (first, second, third, fourth and fifth 10-repetitions), compared each block, and noted a decrease in the instantaneous median frequency as the protocol progressed. They also indicated that SPM showed particular time-regions where the fatigue effect was observed (Gaudet et al., 2018b). In particular, using SPM, it was revealed that signs of fatigue were primarily evident when muscles were acting as the agonist, while the traditional statistical method could not detect meaningful changes, which shows the strength of SPM analysis (Gaudet et al., 2018b). In swimming, observing shoulder muscle activities is highly important from the injury prevention perspective. For example, it has been reported that high levels of swimming training might overload soft tissue structures around the shoulder and lead to pain, dissatisfaction, and disability (i.e., swimmers’ shoulder) (Struyf et al., 2017). Moreover, Drigny et al. (2020) reported that high-level swimming practice induced a significant decrease in internal/external and concentric/eccentric functional motion during a competitive season, which might lead to a greater risk of shoulder injury over time (Drigny et al., 2020). However, as highlighted in the present review, the number of studies that focuses on swimmers’ muscles using SPM analysis is limited, meaning that focusing on this topic would be beneficial to provide further insights into the injury prevention of swimmers.

The study of EMG in swimming also debated the use of the coefficient of variation (CV = one standard deviation/mean ∙ 100, in %) to understand the signals’ variability as a discrete variable (Martens et al., 2016). However, as the CV is influenced by the mean value, it was reported that it might overestimate variability in moments in which the muscle is inactive or its activity is weak (Hug et al., 2008; Martens et al., 2016). Conversely, EMG signals analyzed through continuous analysis (such as SPM) were found to: i) present deeper insights about the inter-variability in muscle recruitment (Martens et al., 2016; Gaudet et al., 2018a; Blache et al., 2018), and; ii) allowed to observe that changes in median decline frequency only occurred in certain time-regions being different across muscles (Gaudet et al., 2018b). Notwithstanding, it must be mentioned that the protocols used in the studies were performed on dry-land rather than in-water.

Regarding swimming velocity, four studies were included in this review (Gonjo and Olstad, 2021; Ruiz-Navarro et al., 2021; Gourgoulis and Nikodelis, 2022; Morais et al., 2023). The studies by Gonjo and Olstad (2021), and Gourgoulis and Nikodelis (2022) were related to breaststroke. The former tested differences in swimming velocity in a set of key-moments between groups (elite vs. sub-elite) (Gonjo and Olstad, 2021), and the latter tested differences in swimming velocity in several key-moments between maximal and sub-maximal trials (Gourgoulis and Nikodelis, 2022). As mentioned earlier, research in swimming puts a lot of focus on understanding the swimming velocity determinants (Andersen et al., 2021; Morais et al., 2022a; Olstad et al., 2022). However, even when the instantaneous velocity is measured, researchers commonly use the average value of the stroke cycle (or the combination of several stroke cycles) (Morais et al., 2018; Silva et al., 2019). It may be suggested that this is done because it is easier for data handling and because researchers might not be aware of statistical packages that allow handling 1-D variables (such as swimming velocity) (Morais et al., 2022c). Discrete variables (0-D) do not verify differences in a specific moment of a given swim stroke because only the average value of the entire stroke cycle can be used. Conversely, by using SPM, it was possible to identify the specific key-moment(s) of a given swim phase in which elite and sub-elite breaststrokers were different (Gonjo and Olstad, 2021). However, it was also noted that the consideration of changes/differences in the duration of movement phases is essential in SPM because some differences between maximal and sub-maximal trials in breaststroke were mainly related to the adjustment of the duration of the glide phase rather than the real changes in the kinematic variables tested in each phase (Gourgoulis and Nikodelis, 2022).

The study by Ruiz-Navarro et al. (2021) was related underwater undulatory velocity. Recently, a review study summarized the state-of-art on this topic by: i) synthesizing the scientific evidence on the kinematic determinants of competitive swimmers during underwater undulatory swimming, and ii) summarizing the main methodological considerations for underwater undulatory swimming kinematic analysis (Veiga et al., 2022). The authors claimed that no solid conclusions were retrieved for the relationship between the kinematic determinants analyzed and the underwater undulatory performance (Veiga et al., 2022). This consideration was based on the mixed findings observed in the studies analyzed for the same variable. Nonetheless, based on discrete variables, it was reported that the angular displacement of the lower trunk in the acceleration and deceleration phases was a determinant for a fast average horizontal velocity of the greater trochanter (Ikeda et al., 2021). The study by Ruiz-Navarro et al. (2021) analyzed the effect of training on underwater undulatory velocity using both discrete and continuous variables (SPM). Based on discrete variables, the authors noted that the underwater undulatory velocity significantly increased overall (average, minimum, and peak), but not the kick frequency (Ruiz-Navarro et al., 2021). Using continuous variables, the authors provided further details related to these improvements. By SPM, it was observed that from the beginning to nearly 15% of the cycle time and from nearly 50% to the end of the cycle time, the underwater undulatory velocity was significantly faster after the training program than before (Ruiz-Navarro et al., 2021). This allowed researchers to specifically identify where in the entire cycle the training program applied had a significant effect. Indeed, the advantages of these specific identifications were also reported by Morais et al. (2023), but for the front-crawl in young swimmers. The study compared swimming velocity during a complete stroke cycle in young swimmers (both sexes) as a discrete variable and as a continuous variable (SPM) having different performance levels. Swimming velocity presented a significant sex and tier effect, as well as a significant sex*tier interaction as a discrete variable. However, the dv (which represents the velocity fluctuation as a discrete variable measured as being the coefficient of variation) presented a significant tier effect, but non-significant sex effect and sex*tier interaction. On the other hand, SPM revealed that significant sex and tier effects were observed throughout the entire stroke cycle. As for the sex*tier interaction significant differences were observed between ∼6 and ∼18%, ∼62 and ∼72%, and ∼88 and ∼92% of the stroke cycle. Indeed, it was recently argued that the use of the coefficient of variation can deliver misleading insights about the swimmers’ velocity fluctuation (Gonjo et al., 2023). Consequently, the use of 1-D statistical methods such as SPM will allow to better understand the velocity fluctuation over the stroke cycle.

Finally, one study included in this review was about propulsion in front-crawl (Morais et al., 2022c). Nowadays, propulsion in swimming (namely, in front-crawl) is experimentally measured based on pressure sensors (Koga et al., 2020; Kadi et al., 2022). Once again, researchers commonly use discrete variables such as the average propulsion of each hand to understand the amount of force generated in the entire stroke cycle (Koga et al., 2020; Santos et al., 2021). The study by Morais et al. (2022c) aimed to understand if there was a significant difference in propulsion between male junior and juvenile swimmers during a maximal trial at front-crawl. The authors noted a significant difference in swimming velocity between groups. Conversely, swimmers of both groups did not significantly differentiate in propulsion during the entire arm-pull in both upper-limbs (juniors presented greater but not significant propulsion levels). Notwithstanding, based on SPM it was possible to identify where within the arm-pull swimmers had greater differences (despite non-significant). If significant differences had been noted, it would be possible to exactly know where within the entire arm-pull. This information would be of major importance for coaches and swimmers to understand in which moment of the arm-pull propulsion can be maximized to improve swimming performance.

The studies included in this review enhanced the understanding of the outputs that could be retrieved from SPM analysis in swimming. In an EMG context, some findings verified in SPM are not possible to be discovered with traditional 0-D statistical methods. Overall, it was shown that the deltoid medialis and rectus abdominis played determinant roles in different phases of the front crawl arm-pull (Martens et al., 2016). Swimmers with a higher competitive level or more experience were more like to suffer alterations in the shoulder motion (Blache et al., 2018). The muscle recruitment during front crawl revealed significant effects for the velocity and type of contraction in all muscles analyzed, and the supraspinatus and infraspinatus muscles behave more as prime movers than stabilizers (Gaudet et al., 2018a). Finally, SPM indicated that changes in instantaneous median frequency only occurred over certain time-regions and differently across muscles (Gaudet et al., 2018b). Based on the specific outputs that can be retrieved from these studies, it was suggested that in future studies about front crawl, the key features to be analyzed could be even more specifically defined. That is, SPM provides deeper insights about significant differences (i.e., why/how the significant differences in discrete variables were generated) rather than just discussing if there is a difference or not. Studies about swimming velocity (breaststroke, freestyle, and underwater undulatory velocity) and propulsion (front-crawl) also highlighted the SPM advantages in comparison to traditional statistical methods. Once again, SPM allowed identifying more accurately within the stroke cycle where those differences occur. However, when conducting SPM, it is important to consider changes/differences in the phases of the movement between conditions/groups. Notwithstanding, it must be mentioned that only nine studies about swimming velocity were included in this review. At least in dry-land sports (Colyer et al., 2018; Brindle et al., 2020) where velocity-time series can be analyzed it seems that literature provides more evidence about the benefits that this analysis provides. Thus, as well as in dry-land sports or movements, using SPM in swimming (whenever suitable and appropriate) may provide coaches and athletes with more precise and accurate information about where in the stroke cycle swimmers can improve. Researchers may not only focus on swimming velocity during the stroke cycle. They can also focus and get new insights in other phases of a swimming event, such as the start and turn. Indeed, it was noted that PCA was applied in the swimming turns (Burkhardt et al., 2020). All studies included in this review were mainly related to biomechanics, but one can argue that EMG data can also be considered within the physiological topic (Kamen and Caldwell, 1996). Nonetheless, researchers about swimming performance must be aware that SPM can also be used to gather insights about other physiological variables as it was done per example in cycling (Dobler et al., 2022). Based on SPM, the authors revealed important insights about the power output between two different pacing exercises, i.e., a standard-paced and a consistently all-out-paced 3-min cycle time trial. Being both a time-based sport with several race distances, these physiological insights can and should also be applied in a swimming context where the power generated by the swimmers plays a key-role on their performance. Limitations in SPM must also be addressed. Despite being an accurate method to detect two or more signals that have similar patterns but different amplitude some cautions must be taken. When the analysis involves signals with different patterns, then the outcomes should be treated very carefully as there is no guarantee you are comparing the same phases of the motion.

## 5 Conclusion

The articles retained in this systematic review described the advantages of applying SPM in swimming whenever possible. In all variables analyzed (EMG, swimming velocity, and propulsion), SPM outputs provided findings that cannot be detected by discrete variables. Curiously, only four studies were related to swimming velocity, which is considered the key-factor in swimming. Thus, researchers are advised to apply SPM (or other 1-D statistical methods) in time-series data when possible.

## Data Availability

The original contributions presented in the study are included in the article/supplementary material, further inquiries can be directed to the corresponding author.
